# The aryl hydrocarbon receptor promotes aging phenotypes across species

**DOI:** 10.1038/srep19618

**Published:** 2016-01-21

**Authors:** Anna Eckers, Sascha Jakob, Christian Heiss, Thomas Haarmann-Stemmann, Christine Goy, Vanessa Brinkmann, Miriam M. Cortese-Krott, Roberto Sansone, Charlotte Esser, Niloofar Ale-Agha, Joachim Altschmied, Natascia Ventura, Judith Haendeler

**Affiliations:** 1 Central Institute of Clinical Chemistry and Laboratory Medicine, Medical Faculty, University of Duesseldorf, 40225 Duesseldorf, Germany; 2 IUF-Leibniz Research Institute for Environmental Medicine, 40225 Duesseldorf, Germany; 3 Division of Cardiology, Pulmonary Diseases, Vascular Medicine, Medical Faculty, University Duesseldorf, 40225 Duesseldorf, Germany

## Abstract

The ubiquitously expressed aryl hydrocarbon receptor (AhR) induces drug metabolizing enzymes as well as regulators of cell growth, differentiation and apoptosis. Certain AhR ligands promote atherosclerosis, an age-associated vascular disease. Therefore, we investigated the role of AhR in vascular functionality and aging. We report a lower pulse wave velocity in young and old AhR-deficient mice, indicative of enhanced vessel elasticity. Moreover, endothelial nitric oxide synthase (eNOS) showed increased activity in the aortas of these animals, which was reflected in increased NO production. *Ex vivo*, AhR activation reduced the migratory capacity of primary human endothelial cells. AhR overexpression as well as treatment with a receptor ligand, impaired eNOS activation and reduced S-NO content. All three are signs of endothelial dysfunction. Furthermore, AhR expression in blood cells of healthy human volunteers positively correlated with vessel stiffness. In the aging model *Caenorhabditis elegans*, AhR-deficiency resulted in increased mean life span, motility, pharynx pumping and heat shock resistance, suggesting healthier aging. Thus, AhR seems to have a negative impact on vascular and organismal aging. Finally, our data from human subjects suggest that AhR expression levels could serve as an additional, new predictor of vessel aging.

Epidemiological studies on populations heavily exposed to 2,3,7,8-tetrachlorodibenzo-p-dioxin (TCDD) like 1976 in Seveso indicate increasing development of cardiovascular diseases in these populations^1^. Moreover, dioxin-exposed workers display higher incidence in atherosclerotic plaques and increased intima-media wall thickness^2^, a risk factor and predictor for stroke and myocardial infarction^3^. TCDD is a ligand for the aryl hydrocarbon receptor (AhR), an intracellular chemosensor belonging to the basic helix–loop–helix/Per-ARNT-Sim family of transcription factors. In the absence of a ligand, AhR rests in a cytosolic multiprotein complex including the heat shock protein 90 (HSP90)^4^. Upon ligand binding, the AhR sheds its co-chaperones, translocates into the nucleus, dimerizes with Aryl Hydrocarbon Receptor Nuclear Translocator (ARNT) and enforces transcription via binding to xenobiotic-responsive elements in the promoters of target genes. The genes activated by AhR code for drug metabolizing enzymes (e.g. cytochrome P450 1A1) as well as for proteins involved in regulation of cell growth, differentiation and apoptosis^5^. The AhR is highly conserved in phylogeny from invertebrates to vertebrates. It is not only involved in drug metabolism but also plays important roles in the regulation of certain physiological processes. This implies that intrinsically produced ligands for this receptor exist and several endogenous compounds have been identified, which are able to modulate AhR activity, however, their physiological relevance is unclear (for review see^6^).

AhR is constitutively expressed in many adult, mammalian tissues with the highest levels of mRNA detected in liver, kidney, lung and heart^7^,^8^. The physiological role of the AhR was studied in three independently generated strains of AhR-deficient mice. The only consistent phenotype is a reduced liver size during development, which disappears between 2 and 4 weeks of age^9^,^10^. Lahvis *et al.* reported that one possible mechanism for this phenomenon is inefficient closure of the ductus venosus within the first two days of life in AhR-deficient mice^11^. Thus, some neo-/perinatal vessel changes occur in AhR-deficient mice. However, for adult animals under standard housing conditions it was assumed that they do not show a vessel phenotype. Atherosclerosis is considered an age-related, chronic inflammatory disease and activation of AhR by its ligands dioxin or benzo[*a*]pyrene (BaP)^10^, a constituent of cigarette smoke, has been shown to promote atherosclerosis^12^,^13^. Consistent with these observations apolipoprotein E knockout mice engineered to express an AhR with 10-fold higher affinity to BaP display bigger hearts under basal conditions, and increased numbers of atherosclerotic plaques in response to BaP when compared to a congenic mouse strain expressing an AhR with lower affinity^14^. Another AhR knockout mouse on a different genetic background shows an increase in medial thickness and a higher number of vascular smooth muscle cells in the aorta^15^. Thus, the AhR could play a role in vascular aging and diseases, since atherosclerosis is seen as an age-related pathology of the vessels.

Therefore, it is important to investigate the role of the AhR in healthy aging and to investigate whether assessment of AhR expression and functionality can tell us something about the biological age of vessels and thereby about the risk for the development of vascular diseases with age. However, up to now there are no experimental data linking the AhR to the functionality of vessels, which decreases with age. Establishing such a connection could potentially provide a new predictor for vascular aging and disease.

In this study we report a decrease in vascular stiffness in AhR-deficient mice with a concomitant increase in the activity of eNOS and NO produced by this enzyme. Along the same lines, we show that AhR activation leads to a reduction of eNOS activity and migratory capacity in human primary endothelial cells (EC), features associated with endothelial dysfunction. Similarly, overexpression of AhR also reduced eNOS activity and S-NO content. Moreover, we demonstrate a positive correlation between AhR expression in blood cells and vessel stiffness in healthy human subjects indicative of vascular aging. In *Caenorhabditis elegans* (*C. elegans*), a widely accepted model for organismal aging, AhR-deficiency led to increased mean life span, motility and stress resistance. In summary, this leads to the conclusion that AhR seems to have a profound negative impact on healthy aging as well as on the cardiovascular system and that AhR expression might be a useful, additional marker for vessel functionality in the prediction of age-associated cardiovascular diseases.

## Results

**Figure 1:**
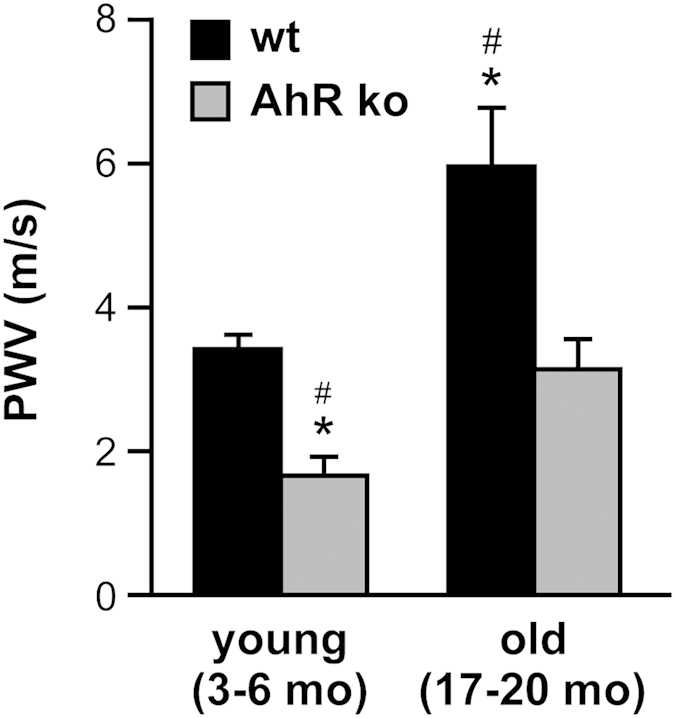
Age-related functional vessel parameters are changed in AhR-deficient mice. PWV in young and old AhR-deficient mice (AhR ko, grey bars) and their wildtype littermates (wt, black bars) was measured as described in the methods section. Young and old AhR-deficient mice show significantly decreased PWV compared to their aged-matched wildtype littermates (mean ± SEM, n = 4–8 per group, *p < 0.05 vs. wt young, ^#^p < 0.05 vs. AhR ko old, unpaired Student’s t-test).

**Figure 2:**
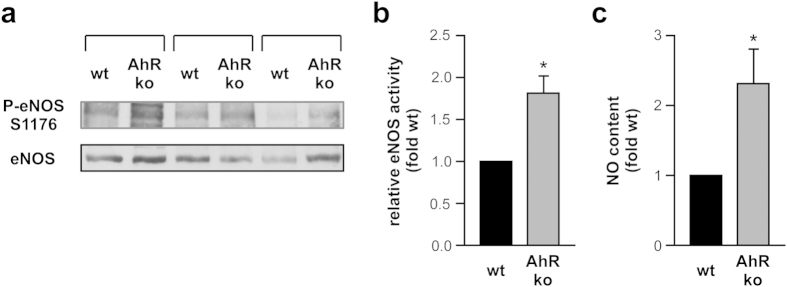
Aortas from AhR-deficient mice show increased active eNOS phosphorylation and NO content. Lysates from thoracic aortas of AhR-deficient mice (AhR ko) and their wildtype littermates (wt) were prepared as described in the methods section and used for immunoblots with anti-phospho serine 1177 eNOS, which recognizes the analogous phosphorylation on serine 1176 in the mouse protein (P-eNOS S1176), and anti-eNOS (eNOS) antibodies and to determine the NO content. (**a**) Immunoblots, bracketed samples represent littermates. (**b**) Semiquantitative analysis, relative eNOS activity is the ratio of P-eNOS S1176 normalized to eNOS (**c**) NO content, wt animals were set to 1 (all data are mean ± SEM, n = 3 per group, *p < 0.05 vs. wt, unpaired Student’s t-test).

The link between environmental toxicants activating the AhR and increased incidence of age-related cardiovascular diseases prompted us to directly assess the role of AhR in vascular aging, which is associated with a number of structural and functional alterations of the endothelium^16^. Among the observed changes are a decrease in the release of vasodilators, an increased synthesis of vasoconstrictors, hypertrophy, the accumulation of extracellular matrix components, and reduction in NO-bioavailability (for review see^17^,^18^). These changes lead to an increase in vascular stiffness, which can be monitored by measuring pulse wave velocity (PWV). An increase of PWV in the elderly^19^ correlates with age and has been suggested as a predictor for future cardiovascular events in healthy subjects as well as in cardiovascular pre-stressed patients^18^. Using a technique previously established by us^20^, we measured PWV in adult (3–6 months) and old (17–20 months) AhR-deficient mice and their wildtype littermates (Fig. 1). As expected, the wildtype animals showed an increase in PWV with age. Interestingly, AhR-deficient mice displayed reduced PWV in young and old animals suggesting reduced stiffness of arteries and thus healthier vessels. Several mechanistic studies *ex vivo* and *in vivo* have demonstrated that expression and/or activity of the NO-producing enzyme eNOS are reduced with increasing age and in disease^21^,^22^ and that decreased phosphorylation of eNOS contributes to age-related vascular stiffness^23^. Thus, we next determined the activity of eNOS, which is reflected by phosphorylation on serine 1176 (mouse), in the thoracic aortas of wildtype and AhR-deficient mice. Congruent with the PWV data, AhR-deficient mice showed a significant, roughly twofold increase in eNOS phosphorylation (Fig. 2a,b). To further support these findings, we measured NO in the aortas after its conversion to nitrite using the Griess assay. As shown in Fig. 2c, the NO content is significantly increased in AhR-deficient animals.

**Figure 3:**
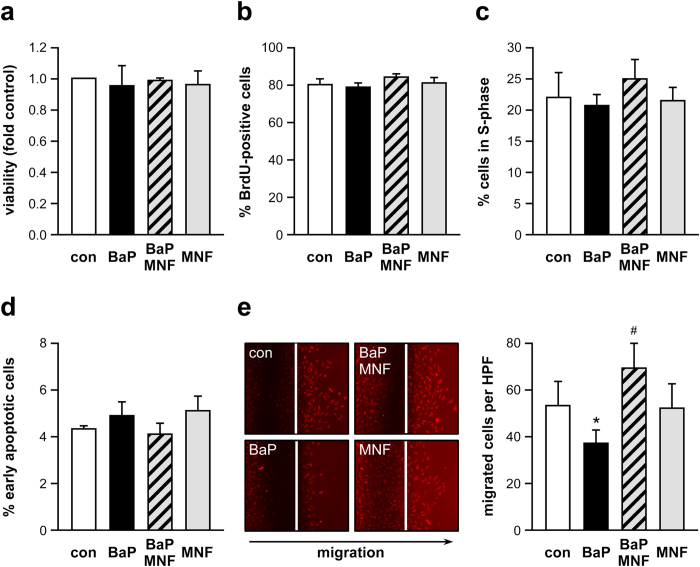
Activation of AhR reduces the migratory capacity of human endothelial cells without affecting proliferation and apoptosis. EC were treated for 18 h with 250 nM BaP (BaP), 2.5 μM MNF (MNF), a combination of both (BaP/MNF) or left untreated (con) and assayed for viability, proliferation, cell cycle phases, apoptosis and migration as described in the methods section. (**a**) Viability: the viability of untreated cells was set to 1. (**b**) Proliferation: shown is the percentage of BrdU-/7-AAD-double positive cells. (**c**) Cell cycle analysis: shown is the percentage of cells in S-phase. (**d**) Apoptosis: annexinV-positive/7-AAD-negative cells are denominated early apoptotic cells. (**e**) Migration: the microscopic pictures show examples of scratch wound assays, the white lines show the origin of migration, the direction of migration is indicated below. The bar graph represents the number of migrated cells per high power field (all data are mean ± SEM, n = 3–4, *p < 0.05 vs. con, ^#^p < 0.05 vs. BaP, paired Student’s t-test).

Given the pronounced negative effect of the presence of AhR on eNOS and NO production in murine aortas, we assessed whether AhR activation has an impact on this enzyme and NO-mediated functions in the human endothelium. Since the functionality of the large vessels depends on the capacity of endothelial cells to proliferate, migrate and to protect themselves against apoptosis, we analyzed the dependence of these parameters on AhR activation in primary human endothelial cells (EC). Therefore, these cells were treated with BaP, a potent activating ligand of AhR and 3′-methoxy-4′-nitroflavone (MNF), an AhR antagonist^24^. First, concentrations of BaP and MNF were determined, which did not elicit any cytotoxic response (Fig. 3a). After that, EC were cultivated in the presence of BaP, MNF or a combination of both. The different treatments had no effect on EC proliferation (Fig. 3b) and cell cycle (Fig. 3c). Similarly, there was neither a change in the percentage of early apoptotic cells (Fig. 3d) nor in the fraction of late apoptotic/necrotic cells (data not shown). However, treatment with BaP significantly reduced migration of endothelial cells (Fig. 3e). This inhibitory effect on EC migratory capacity was completely blunted by coincubation with MNF, whereas MNF alone showed no effect.

**Figure 4:**
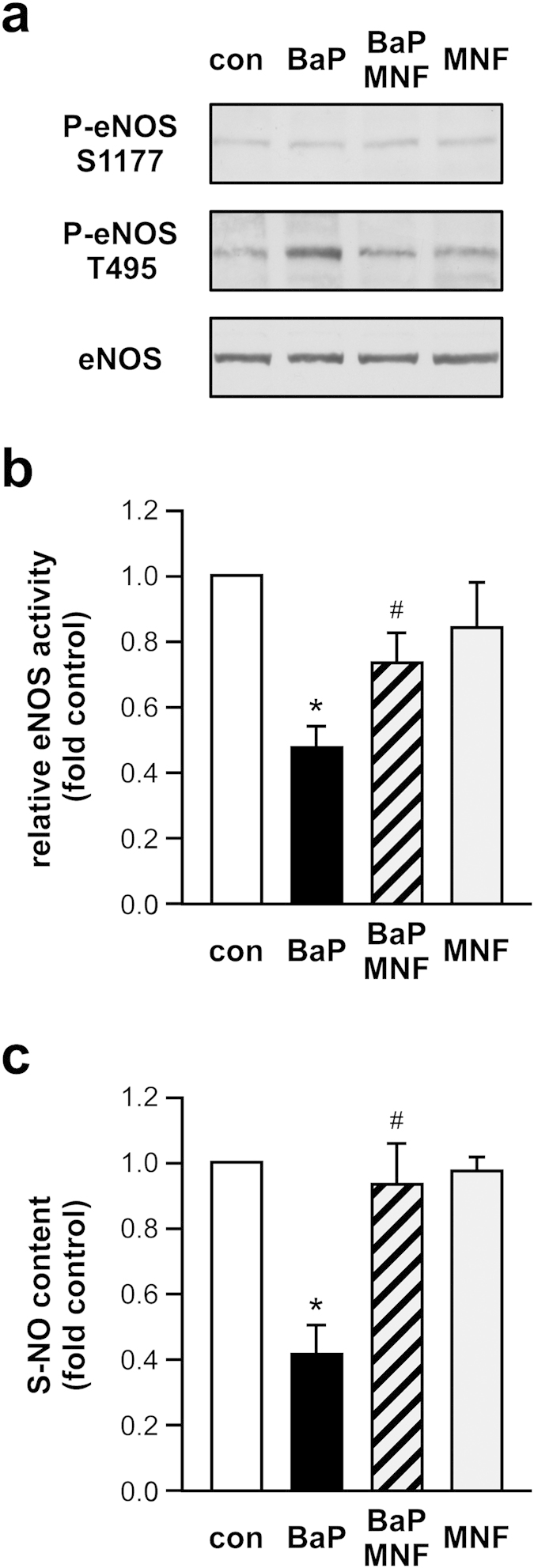
AhR agonist treatment impairs eNOS activation and reduces S-NO content in human endothelial cells. EC were treated for 18 h with 250 nM BaP (BaP), 2.5 μM MNF (MNF), a combination of both (BaP/MNF) or left untreated (con) and lysates were used for immunoblots with anti-phospho serine 1177 eNOS (P-eNOS S1177), anti-phospho threonine 495 eNOS (P-eNOS T495) and anti-eNOS (eNOS) antibodies and determination of S-NO content. (**a**) Representative Immunoblots. (**b**) Semiquantitative analysis, relative eNOS activity is the ratio of P-eNOS S1177 to P-eNOS T495 normalized to eNOS. (**c**) S-NO content, untreated cells were set to 1 (all data are mean ± SEM, n = 3 per group, *p < 0.05 vs. con, ^#^p < 0.05 vs. BaP, paired Student’s t-test).

As the migratory capacity of endothelial cells is dependent on nitric oxide availability, we determined the activation state of eNOS after BaP treatment as the ratio of eNOS phosphorylation on serine 1177 (human/active) and on threonine 495 (human/inactive) as surrogate parameters for enzyme activity. Indeed, incubation of the cells with BaP increased phosphorylation of eNOS on threonine 495. Importantly, coincubation with the AhR antagonist MNF completely abrogated the BaP response, while MNF alone did not affect eNOS phosphorylation (Fig. 4a,b). Activation of eNOS directly correlates with the S-NO content in healthy human endothelial cells and is reduced with age^22^. Therefore, we next measured S-NO content. Indeed, treatment with BaP reduced the S-NO content and this BaP-mediated reduction could be blocked with MNF, which alone showed no effect (Fig. 4c).

**Figure 5:**
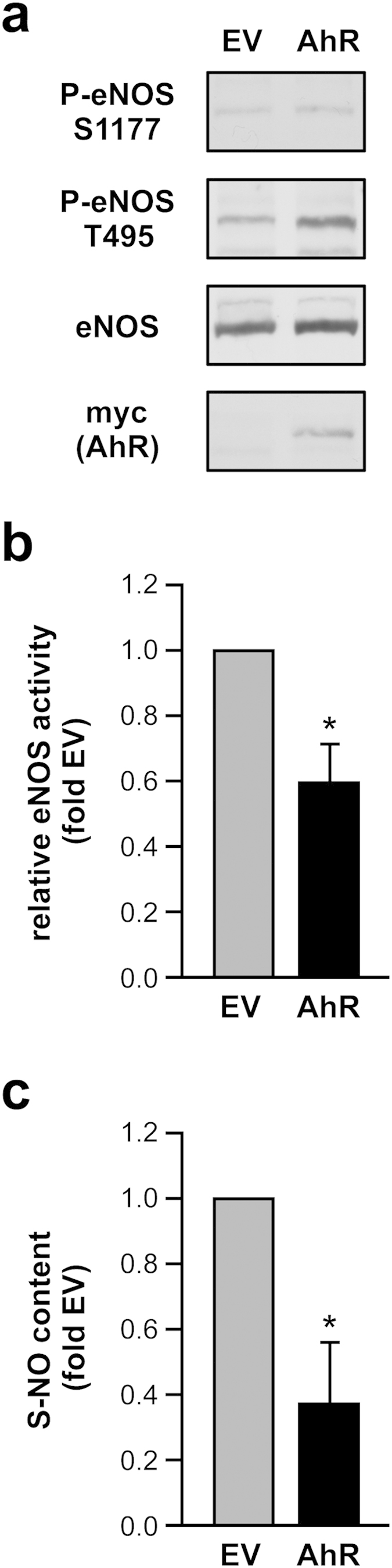
AhR overexpression impairs eNOS activation and reduces S-NO content in human endothelial cells. EC were transfected with an expression vector for human AhR with a C-terminal myc-tag (AhR) or pcDNA3.1/myc-His(-)B as the corresponding empty vector (EV). Lysates were used for immunoblots with anti-phospho serine 1177 eNOS (P-eNOS S1177), anti-phospho threonine 495 eNOS (P-eNOS T495), anti-eNOS (eNOS) and anti-myc tag (myc (AhR) antibodies, the latter to demonstrate expression of exogenously delivered AhR, and for determination of S-NO content. (**a**) Representative Immunoblots. (**b**) Semiquantitative analysis, relative eNOS activity is the ratio of P-eNOS S1177 to P-eNOS T495 normalized to eNOS. (**c**) S-NO content, EV-transfected cells were set to 1 (all data are mean ± SEM, n = 3–4 per group, *p < 0.05 vs. EV, paired Student’s t-test).

In addition to pharmacological activation of AhR, we overexpressed this receptor in EC and again measured eNOS phosphorylation as well as S-NO content. This overexpression resulted in decreased eNOS activation (Fig. 5a,b) and a reduction in S-NO content (Fig. 5c).

Activation and/or overexpression of AhR create situations, which in essence are reciprocal to the absence of functional AhR. Therefore, the effects in primary human endothelial cells together with the observations in the AhR-deficient mice suggest that AhR interferes with vascular functions.

**Figure 6:**
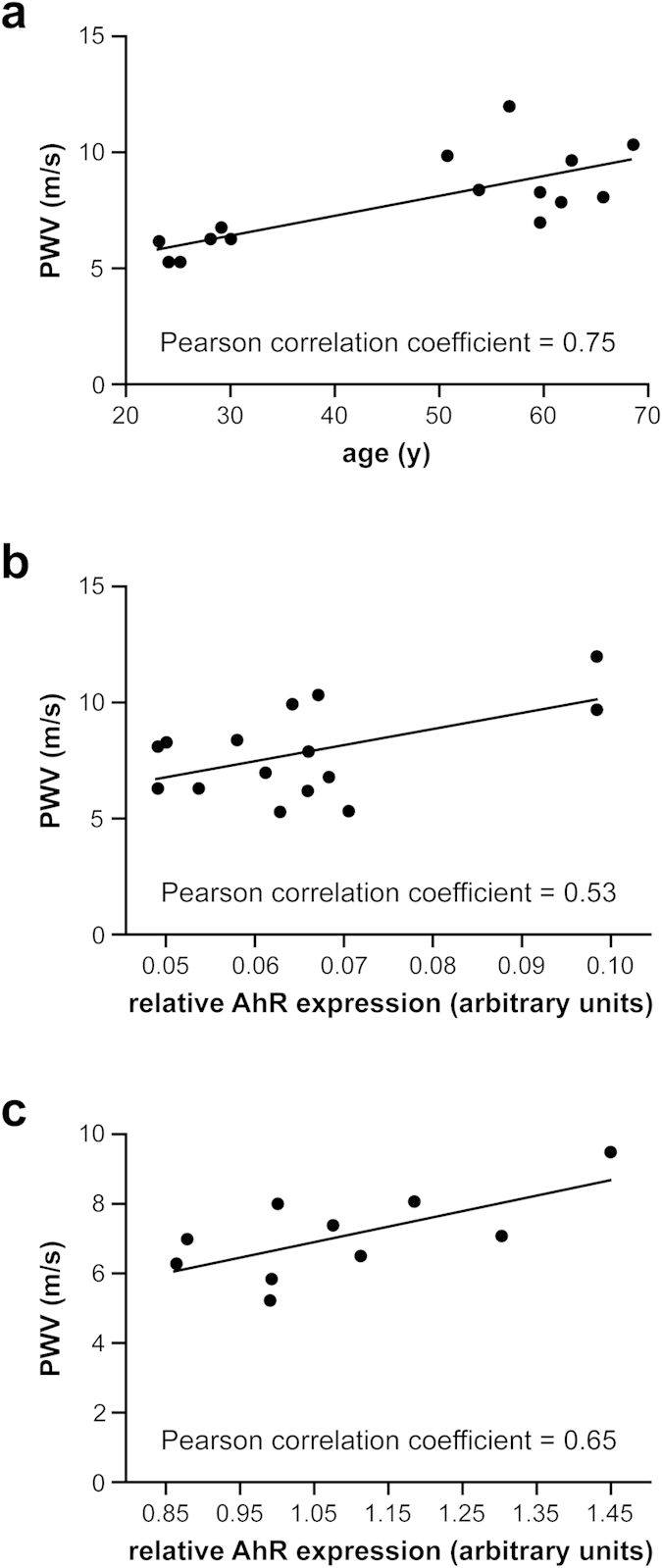
PWV is positively correlated with relative AhR expression in healthy human subjects. (**a**) Correlation between PWV and chronological age in young and elderly subjects (n = 6–9 per group, Pearson correlation coefficient = 0.75). (**b**) Correlation between PWV and relative AhR expression (arbitrary units) in young and elderly subjects (n = 6–9 per group, Pearson correlation coefficient = 0.53). (**c**) Correlation between PWV and relative AhR expression (arbitrary units) in middle-aged subjects (n = 10, Pearson correlation coefficient = 0.65).

To translate our findings regarding the influence of AhR on vessel functionality in mice and *ex vivo* on EC function to human subjects, we determined PWV as a measure for arterial stiffness in young and elderly healthy volunteers (Table 1) and correlated it to the expression levels of AhR in their blood samples. As expected and demonstrated by several groups in large cohort studies^25^,^26^, the PWV strongly and positively correlated with age (Fig. 6a, Pearson correlation coefficient = 0.75). Interestingly, we found a similar correlation between AhR expression and PWV (Fig. 6b, Pearson correlation coefficient = 0.53). To bridge the gap between these two age groups, we analyzed a second collective of healthy individuals (Table 2) in the middle-age range. In this group we also found a positive correlation between AhR expression and pulse wave velocity (Fig. 6c, Pearson correlation coefficient 0.65) strengthening our findings above. Given this correlation, we suggest that the AhR expression level, which can easily be determined in human blood samples, could be used as an additional indicator for vessel functionality and perhaps aging and thus, might be useful in predicting future cardiovascular events.

**Figure 7:**
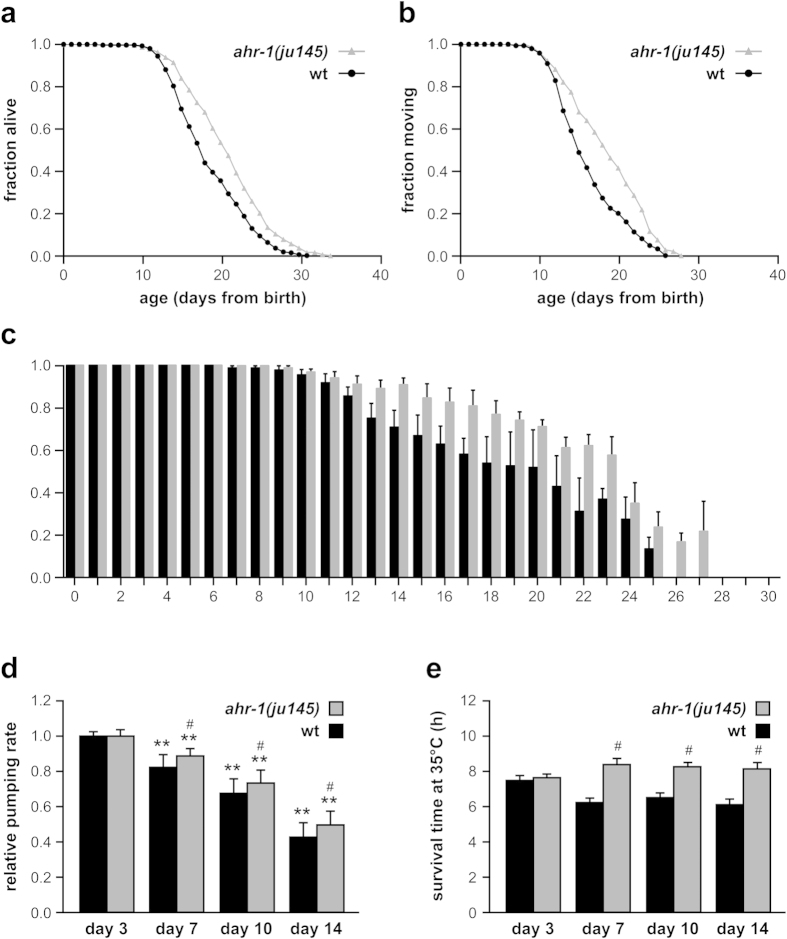
AhR-deficiency extends *C. elegans* healthy life span. (**a**) Life span analysis. Kaplan-Meier survival analysis showing the fraction of animals alive (mean ± SEM, wt = 18.69 ± 0.31, *ahr-1(ju145)* = 21.04 ± 0.36, five independent replicates, animals/censored: wt = 307/78, *ahr-1(ju145)* *=* 299/102, p = 1.8 × 10^−6^ log-rank test between curves). (**b**) Health span analysis. Movement was set as health parameter and the fraction of animals moving was determined (mean ± SEM, wt = 16.30 ± 0.37, *ahr-1(ju145)* *=* 18.74 ± 0.40, three independent replicates, animals/censored: wt = 168/39, *ahr-1(ju145)* *=* 180/32, p = 1.7 × 10^−5^ log-rank test between curves). (**c**) Fraction of animals moving of fraction alive (mean ± SEM, data from animals shown in b). (**d**) Health span analysis. Relative pharynx pumping rate was set as health parameter and the number of pharyngeal bulb contractions was measured at different animal age (mean ± SEM normalized to day 3, three independent replicates, 10 animals/replicate, **p < 0.01 vs. day 3 of same genotype, ^#^p < 0.05 vs. wt same day). (**e**) Health span analysis. Heat stress resistance was set as another health parameter. Survival of animals of different age was assessed at 35 °C (mean ± SEM, three independent replicates, 20 animals/replicate, ^#^p < 0.01 vs. wt same day).

To further determine whether AhR expression is also relevant for organismal aging and healthy life span, we turned to the nematode *C. elegans*, one of the most extensively characterized whole animal models for aging and age-related diseases^27^. The *C. elegans* AhR homolog AHR-1 shares 38% identity with the human protein and is able to bind the nematode ARNT homolog AHA-1 as well as mammalian HSP90^28^. With the aim of addressing the role of AhR in organismal aging, we compared the life span and associated health span phenotypes between wildtype *C. elegans* and the AhR-deficient strain *ahr-1(ju145)*^29^. Life span analysis showed that *ahr-1(ju145)* mutants have a significantly longer mean life span than wildtype animals (Fig. 7a). In *C. elegans*, a typical age-associated phenotype is the decline in locomotion activity^30^. Importantly, we found that loss of *ahr-1* not only extended life span but also significantly improved health span as revealed by the increased fraction of animals capable of moving at any day of life span compared to their wildtype counterparts (Fig. 7b). This difference was even more striking when we only considered the fraction of animals moving among the number of animals still alive at any given day (Fig. 7c).

Although *C. elegans* as an invertebrate has neither a closed circulatory system nor a heart, the animals’ pharynx has been suggested to be an ortholog of the vertebrate heart based on several similarities: Both organs are continuously pumping throughout lifetime and rely on a comparable electrical circuitry. Furthermore, their genesis depends on Nkx transcription factors^31^. Notably, pharynx pumping was recently exploited to study the heart-specific toxicity of human amyloidogenic immunoglobulin light chains from amyloidosis patients suffering from cardiomyopathy^32^. Therefore, we measured the decline in pharynx pumping as an additional, typical age-associated parameter and as a surrogate for mammalian heart function. While wildtype animals showed a progressive decrease in the pharyngeal pumping rate with age, this decline was significantly alleviated in the *ahr-1(ju145)* mutant strain (Fig. 7d).

Another classical parameter that concurrently tracks with *C. elegans* aging is the animals’ decline in resistance to different types of stressors and in particular to heat shock. In this context it is important to mention, that increased expression of heat shock proteins and resistance to heat stress positively correlate with *C. elegans* life span extension in different longevity mutants being indicative of better health^33^. We therefore assessed the survival time of wildtype and AhR-deficient animals of different age at elevated temperature. While, as expected, heat resistance declined in wildtype animals older than 3 days, aged *ahr-1(ju145)* mutants were significantly more resistant to heat shock than their age matched wildtype counterparts (Fig. 7e).

Collectively, these data clearly indicate that loss of AhR extends healthy life span in *C. elegans* supporting the data obtained in mice and humans.

## Discussion

The major findings of the present study are that aryl hydrocarbon receptor deficiency results in improved vessel functions in mice and enhanced healthy life span in *C. elegans* and, furthermore, that activation of the receptor suppresses endothelial cell migration, similar to what is observed in the aged vasculature. Furthermore, activation or overexpression of AhR in EC interferes with eNOS activation and S-NO content. In addition, we find a positive correlation between AhR expression and vessel stiffness in healthy human subjects.

It has been shown that the cardiovascular system of congenic mouse strains expressing AhRs with different responsiveness to BaP is differentially affected by this AhR ligand^14^. Furthermore, another AhR-knockout strain on a different genetic background shows thickening of the arterial media and increased numbers of vascular smooth muscle cells in the arterial wall^15^. However, a direct role for AhR in cardiovascular and organismal aging has never been demonstrated across species from invertebrates to humans.

Our initial observation of a lower pulse wave velocity and thus, decreased vessel stiffness in AhR-knockout mice can possibly be ascribed to the enhanced phosphorylation of murine eNOS on serine 1176, a surrogate parameter for the activation of this enzyme and the concomitant increase in NO production. Although vascular stiffness has traditionally been attributed to structural changes in the vessel wall, i.e. remodeling of the extracellular matrix, it has become evident that smooth muscle cells contribute to arterial stiffening during aging^34^. This goes along with the fact that smooth muscle tone is regulated by endothelium-derived NO and the observations that a decreased NO production or inhibition of eNOS can contribute to vascular stiffness^23^. In line with the enhanced activation of eNOS and NO production in the aortas of the AhR-deficient mice, we found an increase in the inactivating phosphorylation of eNOS on threonine 495 in human endothelial cells after treatment with the AhR ligand BaP, which was accompanied by a decrease in S-NO content, a response that could be blunted by coincubation with the antagonist MNF. Both, the *in vivo* and *ex vivo* observations go along with a report showing that AhR activation induces senescence in endothelial cells^35^, which among other features is characterized by reduced NO-bioavailability^22^. The nature of AhR as a transcription factor is suggestive of an involvement of AhR target genes and is has been shown that aging-associated gene expression profiles are altered in hematopoietic stem cells of AhR-deficient mice^36^. However, the observed change in the post-translational modification of eNOS could only indirectly be affected by changes in transcription rates. This leads to the speculation that other mechanisms might be involved in the AhR-dependent impairment of vascular and endothelial functions. A potential direct link between AhR and eNOS is HSP90. Activation of eNOS is dependent on the association with HSP90^37^ and the eNOS/HSP90 complex has been demonstrated to be responsible both for serine phosphorylation and threonine dephosphorylation of eNOS^38^. Since HSP90 is also a chaperone for AhR^4^ and the expression level of HSP90 in endothelial cells is reduced with age^39^, one could speculate that the improvement of vascular function in aged AhR-deficient mice, which is accompanied by an enhanced eNOS activation, is a result of increased HSP90/eNOS complex formation due to a lack of competition with AhR. Further circumstantial evidence for this hypothesis is provided by the positive correlation between AhR expression and PWV in the healthy human subjects, which most likely also has an NO-dependent component. In addition, complex formation with HSP90 has also been suggested to prevent eNOS uncoupling^40^. Therefore, it is conceivable that the age-dependent uncoupling of eNOS, which contributes to endothelial dysfunction^41^, is also dependent on AhR expression levels providing a second layer of AhR-interference with eNOS and thus vascular functions.

Moreover, there is emerging evidence that AhR expression is linked to an inflammatory response. Inflammation is known to accelerate vessel dysfunction and aging. It has been demonstrated that AhR activation induces vascular inflammation leading to upregulation of pro-inflammatory genes in macrophages and mouse aortas^13^. Thus, high level AhR expression in the large vessels could contribute to endothelial dysfunction, which is in line with our findings presented here. Moreover, it has recently been shown that TCDD increases inducible nitric oxide synthase (iNOS) expression during influenza viral infection. This requires the DNA binding domain of AhR suggesting that the receptor has to bind to the iNOS promoter to induce expression of the enzyme^42^. Since it has been demonstrated that during endothelial dysfunction and vessel aging, eNOS levels are reduced and iNOS expression is increased^16^, it is tempting to speculate that enhanced iNOS levels in aged vessels are dependent on high AhR expression. Thus, higher AhR expression could result in vascular inflammation through this pathway and thereby further contribute to endothelial dysfunction and vessel aging.

Besides enhancing the inactivating phosphorylation of eNOS on threonine 495, AhR activation with BaP reduced migration of primary human endothelial cells, a process required for angiogenesis. It has been demonstrated that hindlimb ischemia-induced angiogenesis is enhanced in AhR-deficient mice^43^ and inhibited after oral administration of BaP in wildtype animals^44^, underscoring the negative impact of AhR on angiogenesis. One possible explanation is the association of AhR with the transcriptional coactivator CREB-binding protein (CBP) (for review see^45^). Since CBP is required for angiogenesis^46^ and seems to be present in limiting concentrations within cells (for review see^47^) one could speculate that reduced expression or depletion of AhR could increase the level of freely available CBP thus leading to increased migration. CBP is an essential mediator in the response to cyclic AMP (cAMP) via the cAMP-response element-binding-protein (CREB). In support of our hypothesis, it was shown in an experimental stroke model that interference of AhR with this pathway by complex formation with CREB and CBP could be responsible for the deleterious effects of AhR^48^.

In *C. elegans* we did not only observe an increase in mean life span and health span (motility) when comparing AhR-deficient to wildtype animals, but also improved pharynx pumping and heat shock resistance. The molecular mechanisms behind these phenomena are still elusive. AHR-1 deficiency in the *ahr-1(ju145)* mutant leads to adoption of a different fate in GABAergic neurons, which control head muscle movements^29^. However, no alterations in pharyngeal pumping or motility have previously been observed in the *ahr-1* mutant strain. Nevertheless, based on the functions of the *C. elegans* homologs of vertebrate HSP90 and CBP, DAF-21 and CBP-1, respectively, one could speculate that a similar mechanism as in mice and humans, i.e. sequestration of these proteins by AHR-1 and thus, an increased free pool of these proteins in the *ahr-1(ju145)* mutant, is in effect in this nematode.

The heat shock response in *C. elegans* has been linked to life span by showing that a small heat shock protein is upregulated in long-lived mutants^33^. Furthermore, the heat-shock transcription factor 1 (HSF-1) is required for life span extension via the insulin/IGF-1 pathway^49^. HSF-1 controls the induction of multiple heat shock proteins including DAF-21/HSP90^50^. Direct evidence for the role of this heat shock protein in organismal aging has been provided by downregulation of *daf-21* resulting in a reduction of life span^49^. Interestingly, DAF-21 has also been implicated in muscle functionality, as mutation or downregulation of this protein is associated with defects in muscle functionality and motility^51^. As hypothesized before, an increased availability of DAF-21 in the *ahr-1* knockout strain, could thus serve to explain the improved motility, pharynx pumping and heat shock resistance in these animals.

With respect to CBP, there is a single report showing that downregulation of *C. elegans cbp-1* by RNA interference partially blocks the life extending phenotype of the *daf-2* mutation and completely abrogates life span extension by dietary restriction^52^, demonstrating an important role for CBP-1 in aging.

The congruent effects observed in the three species without treatment with substances known to activate AhR support the idea that endogenous ligands exist, which mediate at least some of the functions of this receptor. In this context, it has been speculated that the ancestral role of AhR is unrelated to the known toxin response, as AhR is an ancient molecule, which was already present in the common ancestor of vertebrates and invertebrates. Furthermore, the *C. elegans* receptor does not bind dioxins^28^. A number of intrinsically produced compounds have been shown to modulate AhR activity (for review see^6^) among them modified low density lipoprotein^53^, which might be involved in the effects on the cardiovascular system. This does not imply that AhR is inherently linked to negative outputs for an organism, especially in light of the evolutionary conservation of this protein. It is more likely that its presence and/or activation is required sometime during the life of an individual. However, a better understanding of the actions of putative endogenous and possibly exogenous ligands is required to appreciate the full functional spectrum of AhR actions.

In conclusion, our data suggest that AhR has a negative impact on healthy aging across species. Moreover, given the correlation between PWV and AhR expression in human subjects, one could hypothesize that the expression level of AhR in human blood cells may serve as an additional indicator for the biological age of vessels, which in combination with other markers could help to better predict future cardiovascular events. Further, detailed characterization of AhR-functions in aging and age-related disease might help to uncover the ancient functions of this protein and to develop strategies for healthier aging.

## Methods

### Mice

AhR-deficient mice^54^ on a C57BL/6 background and their wildtype littermates were used. Mice were bred at the animal facility of the IUF and kept under specific pathogen free conditions under a 12 hour light/dark cycle. They had ad libitum access to food and water. The protocol was approved by the governmental committee of animal welfare, environment and consumer protection of North Rhine-Westphalia (LANUV 84-02.04.2011.A229). All methods were carried out in accordance with the approved guidelines.

### Measurement of pulse wave velocity in mice

Pulse wave velocity (PWV) measurements were performed as previously described^20^. Animals were anesthetized with isoflurane. In order to determine PWV, the angle-corrected Doppler velocity spectra were recorded in the femoral artery along with an EKG using high resolution ultrasound probe (Vevo 2200, Visualsonics). The pulse travel time was calculated as the time delay between the R-wave of the EKG and onset of systolic blood flow. PWV was calculated by dividing the distance between the suprasternal notch and the location of Doppler flow measurements in the femoral artery by the pulse travel time. All methods were carried out in accordance with the approved guidelines.

### *C. elegans* strains and maintenance

Animals were kept (semi-)synchronized (by egg lay) at 20 °C on Nematode Growth Media (NGM) plates and fed with *Escherichia coli* OP50. For the experiments worms were synchronized on plates supplemented with *E. coli* HT115. This bacterial strain is used for dsRNA interference and empty-vector transformed bacteria are routinely used nowadays in many laboratories for consistency also in experiments that do not require RNAi feeding. Bacteria were grown in LB medium at 37 °C overnight. *C. elegans* strains used for the experiments were obtained from the Caenorhabditis Genetics Center: N2: wildtype and CZ2485: *ahr-1(ju145)*^29^; the latter was outcrossed more than four times as specified on the CGC website ( www.cgc.cbs.umn.edu/strain.php?id=7760).

### *C. elegans* life span assay

Life span analysis was started from a 3 hours synchronized egg lay and was carried out with 60 animals in 6 cm plates. From the third day after the egg lay the worms were transferred to fresh NGM plates every day during the fertile phase (mainly day 3 – 6) to prevent starvation and contamination by the progeny. Later, the animals were transferred every second or third day. While transferring the number of worms alive and dead was counted. Animals that did not move spontaneously nor responded to a manual stimulus or did not show any sign of pharyngeal pumping were scored as dead. Worms showing an egg laying defect (bags) or an exploded vulva, or that died desiccated on the wall or were lost during the life span, were listed as censored. The life span curve was calculated from the dead and censored animals using a Kaplan-Meier estimator for life span curves and the p values were calculated using the log-rank test between pooled populations of animals.

### *C. elegans* motility assay

To investigate healthy aging in *C. elegans*, movement was set as a health parameter. The fraction of animals, which were either moving spontaneously or which were able to move after a manual stimulus, were counted as moving while dead animals or animals that were not moving or just moving the head, were counted as not moving. Statistical analysis was carried out as described for life span assay.

### *C. elegans* pharyngeal pumping quantification

Pharyngeal pumping was quantified by counting the grinder movements in the terminal bulb using a stereomicroscope (Zeiss Discovery V8, 80x magnification). To this extent, a synchronized population of 20 worms was maintained by transferring to fresh media every other day. On day 3, 7, 10 and 14 the pumping of 10 animals was counted twice per animal for a 20 second interval. Only animals, which were crawling on the bacteria, were counted.

### *C. elegans* resistance to heat stress

Heat stress was performed at day 3, 7, 10 and 14 on 20 animals/time point at 35 °C on 3 cm plates wrapped with parafilm in an incubator (Intrafors HT Multitron). The worms for the experiment were taken from a synchronized population, which, starting from a population of 140 worms, was maintained by transferring to fresh media every day. Survival was assayed every hour by gently touching the worms with a platinum wire. Worms, which were not responding to touch and did not show pharyngeal pumping activity, were counted as dead. The mean survival time was calculated for each day.

### Human subjects

We recruited young (<35 yrs), elderly (>50 yrs) and middle-aged healthy (35–60 yrs) male subjects without history, signs, or symptoms indicative of cardiovascular disease, including previous myocardial infarction, stroke, and peripheral artery disease or current or previous medication. The ethics committee of the Heinrich-Heine-University approved the study protocol, and all subjects gave written informed consent (Clinicaltrials.gov: NCT01639781, NCT01799005). All methods were carried out in accordance with the approved guidelines.

### Measurement of pulse wave velocity in humans

PWV of the subjects was determined by aplanation tonometry using the SphygmoCor® system with pulse travel time measurements taken at the carotid and femoral artery in accordance with the recommendations of the expert consensus on arterial stiffness^55^.

### Gene expression analyses of human whole blood samples by reverse transcriptase PCR

Venous blood samples were taken from the cubital vein into a Pre-Analytix PaxGene RNA tube (Qiagen, Hilden, Germany) and stored at −80 °C until preparation. Total RNA extraction was performed following the manufacturer’s protocol. RNA quality was assessed by an Agilent Bioanalyzer using a Nanochip. Samples not meeting the quality control criteria were discarded. 0.5 μg total RNA was transcribed into cDNA. For amplification we used commercially available TaqMan primers and probes directed against AhR, a housekeeping mRNA, and gene expression master mix (Life Technologies GmbH, Darmstadt, Germany).

### Gene expression analyses of human whole blood samples by microarray hybridization

RNA was isolated using the PAXgene Blood RNA Kit (PreAnalytix GmbH, Hombrechtikon, Switzerland) as recommended by the manufacturer. The quality of obtained total RNA was verified by the Agilent 2100 Bioanalyzer (Agilent, Santa Clara, USA). All samples showed common high quality RNA integrity numbers (RINs) between 7,8 and 10. RNA was quantified by photometric Nanodrop measurement. Synthesis of biotin labeled cRNA, hybridization to Affymetrix PrimeView Human Gene Expression Microarrays and subsequent microarray staining were performed according to the manufacturers´ protocols (3′ IVT Plus Kit; Affymetrix, Inc.). Data analyses on Affymetrix CEL files were conducted with GeneSpring GX software (Vers. 12.5; Agilent Technologies). Probes within each probeset were summarized by RMA after quantile normalization of probe level signal intensities across all samples to reduce inter-array variability^56^.

### Generation of an AhR expression vector

To obtain an expression vector for the human AhR with a C-terminal myc-tag, the AhR coding sequence was amplified from endothelial cell cDNA with primers containing appropriate restriction sites and a Kozak sequence and inserted into pcDNA3.1/myc-His(-)B (Life Technologies GmbH, Darmstadt, Germany). Briefly, total cellular RNA was isolated from endothelial cells using Trizol (Life Technologies GmbH, Darmstadt, Germany) according to manufacturer’s specifications. The concentration was determined spectrophotometrically and the integrity was assessed by agarose gel electrophoresis. 5 μg RNA were reverse transcribed using the SuperScript III First-Strand Synthesis System (Life Technologies GmbH, Darmstadt, Germany) with random hexamers and oligo(dT)_20_ according to manufacturer’s recommendations. Amplification was performed with Velocity DNA polymerase (Bioline, Luckenwalde, Germany). Amplification products were purified with the QIAquick PCR purification kit (Qiagen, Hilden, Germany), digested with appropriate restriction enzyme and, after resolution on an agarose gel, re-purified with the QIAquick gel extraction kit (Qiagen, Hilden, Germany) and ligated with the digested target vector. After transformation into competent *E.coli* TG1 DNA was isolated from single colonies with the QIAprep Spin Miniprep Kit (Qiagen, Hilden, Germany). The identity of the plasmids was verified by restriction analysis and DNA sequencing of the complete AhR coding region. Further details of the cloning procedure are available upon request.

### Cell culture

Human primary umbilical vein endothelial cells (EC) were cultured as previously described^57^. After detachment with trypsin, cells were grown for at least 18 h.

### Transient transfection of endothelial cells

Plasmid DNAs for transfections were purified using the HiSpeed Plasmid Maxi Kit (Qiagen, Hilden, Germany) and EC were transfected using SuperFect Transfection reagent (Qiagen, Hilden, Germany) as previously described^57^.

### Viability assay

Cell viability was analyzed by the MTT (3-(4,5-dimethylthiazol-2-yl)-2,5-diphenyltetrazolium bromide) tetrazolium reduction assay. Cells were seeded in six-well plates at 7 × 10^4^ cells/well. Starting 24 h later, the cells were treated with BaP, MNF or BaP/MNF; DMSO served as negative control. After 18 h of treatment, the cells were washed three times with PBS and incubated with MTT at a concentration of 0.25 mg/ml (500 μg total) in culture medium. After incubation at 37 °C for four hours, the medium was discarded and the formazan product was solubilized by adding 1 ml DMSO. Absorbance was measured at 550 nm in a microplate reader (Infinite200 Pro, Tecan, Männedorf, Switzerland).

### Cell proliferation and cell cycle analysis

Proliferation was measured by the incorporation of 5-bromo-2′-deoxyuridine (BrdU) as a parameter for DNA synthesis using the BrdU Flow Kit (BD Biosciences, Heidelberg Germany) according to manufacturer’s recommendations as previously described^58^. In brief, BrdU was added to the culture medium 18 h before cell detachment. Incorporated BrdU was labeled with a FITC-coupled anti-BrdU antibody according to manufacturer’s protocol and permeabilized cells were counterstained with 7-amino-actinomycin (7-AAD). Cell size and shape were determined by forward and side scatter and gated cells were analyzed for FITC- and 7-AAD fluorescence using a FACSCalibur flow cytometer (BD Biosciences, San Jose, CA, USA). Cells positive for FITC and 7-AAD were classified as BrdU-positive cells. Cell cycle analysis was performed using the FloJo software package; the cell cycle distribution of 7-AAD-positive cells was determined with the Dean-Jett-Fox model.

### Apoptosis assay

Detection of cell death was performed by flow cytometry using annexinV-FITC binding and 7-amino-actinomycin (7-AAD) staining as described previously^57^. Cells positive for annexinV and negative for 7-AAD were classified as early apoptotic cells, double positive cells as late apoptotic/necrotic.

### Migration assay

Migration was quantified with a scratch wound assay as described previously^59^.

### Immunoblotting

Murine thoracic arteries were ground in liquid nitrogen and the resulting powder was lysed in RIPA-buffer (50 mM Tris-HCl pH 8.0, 150 mM NaCl, 1% (v/v) Igepal CA-630, 0.5% (w/v) deoxycholic acid, 0.1% (w/v) sodium dodecyl sulfate) for 30 min on ice. After removing cellular debris by centrifugation (16.000× g, 15 min, 4 °C), protein concentrations were measured using the Bradford reagent (BioRad, Munich, Germany). Endothelial cells were washed with PBS and scraped off the dishes on ice. After centrifugation, the resulting cell pellet was lysed in RIPA-buffer for 30 min on ice. After removing cellular debris by centrifugation (16.000× g, 15 min, 4 °C), protein concentrations were measured using the Bradford reagent (BioRad, Munich, Germany). Immunoblotting was performed with antibodies directed against eNOS (1:500) (Abcam, Cambridge, UK), phospho-eNOS (S1177) (1:500), phospho-eNOS (T495) (1:500) and the myc-tag (1:500) (all Cell Signaling Technology, Frankfurt, Germany). Blotting membranes were incubated with primary antibodies overnight at 4 °C before they were washed and incubated with secondary antibodies according to standard procedures. Detection was performed by enhanced chemiluminescence using the ECL reagent (GE Healthcare, Freiburg, Germany) and standard X-ray films. Semi-quantitative analyses were performed on scanned X-ray films using ImageJ 1.42q^60^.

### Determination of nitrite and S-NO content

NO and S-NO content were measured using the Griess or the modified Saville-Griess assay as described previously^57^. In this assay, NO is converted to its stable product nitrite. In brief, ground aortic tissue or endothelial cells were lysed in Griess-lysis buffer (50 mM Tris-HCl pH 8.0, 150 mM NaCl, 5 mM KCl, 1% (v(v) Igepal CA-630, 1 mM phenylmethylsulfonyl fluoride, 1 mM bathocuproinedisulfonic acid, 1 mM diethylenetriaminepenta acetic acid, 10 mM N-ethylmaleimide) and 80 μg to 100 μg of lysate was incubated with 1% (w/v) sulfanilamide and 0.1% (w/v) N-(1-naphthyl)ethylenediamine dihydrochloride in the presence or absence of 10 mM CuCl_2_ (Cu^2+^-ions) for 20 min. The optical density obtained in the absence of Cu^2+^-ions results from nitrite, whereas the optical density in the presence of Cu^2+^-ions results from the Cu^2+^ -ion-triggered release of NO out of S-NO bonds plus from nitrite).

### Statistics

All values except for the clinical parameters are expressed as mean ± SEM. Statistical analysis of raw data were performed with paired or unpaired Student’s t-test, if not indicated otherwise. Data were normalized after statistical analyses.

## Additional Information

**How to cite this article**: Eckers, A. *et al.* The aryl hydrocarbon receptor promotes aging phenotypes across species. *Sci. Rep.* **6**, 19618; doi: 10.1038/srep19618 (2016).

## Figures and Tables

**Table 1:** Characteristics of young and elderly human subjects.

	young (n = 6)	elderly (nv= 9)	p-value
age (y)	26 ± 3	60 ± 6	<0.001
BMI (kg/m^2^)	24 ± 3	26 ± 3	0.089
creatinine (mg/dl)	1.0 ± 0.2	1.0 ± 0.1	0.724
total cholesterol (mg/dl)	187 ± 53	226 ± 42	0.130
LDL cholesterol (mg/dl)	138 ± 24	156 ± 11	0.428
HDL cholesterol (mg/dl)	49 ± 3	50 ± 8	0.915
triglycerides (mg/dl)	110 ± 60	137 ± 30	0.271
fasting plasma glucose (mg/dl)	94 ± 10	94 ± 8	0.981
HbA1c (%)	5.0 ± 0.2	4.2 ± 2.4	0.485
SBP (mmHg)	120 ± 5	131 ± 14	0.060
DBP (mmHg)	77 ± 9	79 ± 9	0.602
HR (bpm)	56 ± 7	55 ± 6	0.705
CRP (mg/dl)	0.1 ± 0.2	0.1 ± 0.1	0.850
Hb (mg/dl)	15.7 ± 0.8	15.6 ± 1.3	0.842
leukocytes (1000/μl)	6.1 ± 0.8	6.4 ± 1.6	0.717

Values are mean ± SD.

**Table 2:** Characteristics of middle-aged human subjects.

	middle-aged (n = 10)
age (y)	41 ± 7
BMI (kg/m^2^)	25 ± 3
creatinine (mg/dl)	0.9 ± 0.2
total cholesterol (mg/dl)	204 ± 33
LDL cholesterol (mg/dl)	136 ± 34
HDL cholesterol (mg/dl)	54 ± 10
triglycerides (mg/dl)	104 ± 32
fasting plasma glucose (mg/dl)	89 ± 6
HbA1c (%)	5.5 ± 0.3
SBP (mmHg)	135 ± 15
DBP (mmHg)	90 ± 25
HR (bpm)	66 ± 9
CRP (mg/dl)	0.1 ± 0.2
Hb (mg/dl)	15.4 ± 0.4
leukocytes (1000/μl)	6.8 ± 1.9

Values are mean ± SD.
